# Perceptual discrimination in the face perception of robots is attenuated compared to humans

**DOI:** 10.1038/s41598-023-42510-6

**Published:** 2023-10-04

**Authors:** Abdulaziz Abubshait, Patrick P. Weis, Ali Momen, Eva Wiese

**Affiliations:** 1 Italian Institute of Technology, Genoa, Italy; 2 George Mason University, Fairfax, VA USA; 3 Julius Maximilians University, Würzburg, Germany; 4 Air Force Academy, Colorado Springs, CO USA; 5 Berlin Institute of Technology, Berlin, Germany

**Keywords:** Social neuroscience, Psychology, Human behaviour

## Abstract

When interacting with groups of robots, we tend to perceive them as a homogenous group where all group members have similar capabilities. This overgeneralization of capabilities is potentially due to a lack of perceptual experience with robots or a lack of motivation to see them as individuals (i.e., *individuation*). This can undermine trust and performance in human–robot teams. One way to overcome this issue is by designing robots that can be individuated such that each team member can be provided tasks based on its actual skills. In two experiments, we examine if humans can effectively individuate robots: Experiment 1 (n = 225) investigates how individuation performance of robot stimuli compares to that of human stimuli that either belong to a social ingroup or outgroup. Experiment 2 (n = 177) examines to what extent robots’ physical human-likeness (high versus low) affects individuation performance. Results show that although humans are able to individuate robots, they seem to individuate them to a lesser extent than both ingroup and outgroup human stimuli (Experiment 1). Furthermore, robots that are physically more humanlike are initially individuated better compared to robots that are physically less humanlike; this effect, however, diminishes over the course of the experiment, suggesting that the individuation of robots can be learned quite quickly (Experiment 2). Whether differences in individuation performance with robot versus human stimuli is primarily due to a reduced perceptual experience with robot stimuli or due to motivational aspects (i.e., robots as potential social outgroup) should be examined in future studies.

## Introduction

When interacting with nonhuman entities like robots or avatars, we tend to overlook their individual characteristics and capabilities, perceiving them as a uniform group^1^. This lack of differentiation stems from limited experience or motivation to process them individually, resulting in reduced perceptual discrimination (i.e., *individuation*^2^). However, in human–robot teams, recognizing the uniqueness of each robot member is crucial for maintaining trust and team performance^3,4^. Failing to do so leads to assumptions about the entire group based on experiences with only one member^3^. Positive experiences may lead to overreliance on all robots, while negative experiences can create widespread distrust^5^. This generalization, known as System-Wide Trust (SWT), harms teamwork by affecting performance and overburdening human operators^2,3^. One way to improve trust calibration is by perceiving the unique capabilities of each robot and avoiding generalizations (i.e., Component-Specific Trust; CST). CST fosters better performance and attitudes in human–robot teams by differentiating agents and developing agent-specific beliefs^1,2^.

But to generate CST, one must be able to reliably individuate robot agents. Fortunately, extensive research has examined the causes of what makes us see agents as unique versus interchangeable. Indeed, an established phenomenon is the tendency to see members of unfamiliar groups as more similar than they actually are (i.e., *outgroup homogeneity*^5–7^). Outgroup homogeneity occurs when people focus attention on category-specifying agent features (i.e., features that an agent has in common with most other group members) rather than individuating features of others (i.e., features that distinguish an agent from other group members^2^). For humans, the most important cues for individuation are derived from their face. These cues include face-variant information reflecting an individual’s internal states (i.e., emotions, intentions), as well as face-invariant information related to an individual’s identity (e.g., gender, age, ethnicity), and there are human brain areas that specifically processes this information^8–10^. In line with the neural specialization, cognitive processing of human faces is also unique compared to other stimuli: whereas most visual stimuli are processed via a piecemeal integration of separate features, faces are processed *configurally*, that is: as an integrated Gestalt (see^11^ for a review). Configural processing is associated with a variety of unique effects and causes perceivers to be sensitive to the orientation in which faces are presented. For instance, when seeing upright human faces, they are processed configurally, which supports strong face encoding and recognition when seeing familiar faces. When human faces are presented upside-down, however, configural processing is disrupted and the recognition of familiar or previously seen faces is significantly impaired (e.g.,^12^). Most importantly, this “inversion effect” does not occur for non-face stimuli, such as objects or animals, which are not encoded configurally but rather processed in terms of their individual features^13,14^; thus, the orientation of presented objects does not matter for recognition.

Since configural processing is positively correlated with face recognition performance, it is not surprising that it enables observers to efficiently individuate human faces (e.g.,^15,16^); objects or non-face stimuli, in contrast, are usually not individuated as well as human faces^17^. Individuation is studied empirically using a paradigm that consists of two phases: in the first phase—the learning phase—participants learn to associate a set of face stimuli with a set of letter identifiers (one identifier per face). In the second phase—the identification phase—participants are shown the same face stimuli again (one at a time) and are asked to pick the specific identifier that corresponds to the presented stimulus. As humans are exposed to faces since birth, it was necessary to create a set of unfamiliar stimuli that were not already overlearned (the *Greebles*) but possessed features (i.e., dunths, boges and quiffs) that were similar to human facial features to justify their use to study face perception^16^. Using these stimuli, it was shown that although it was initially difficult to perceptually discriminate between Greebles, individuation performance improved as participants’ perceptual experience with the stimuli increased. Increased individuation performance was associated with stronger activation in brain areas that later became known as the face perception network including the Fusiform Face Area (FFA) and the Anterior Temporal Lobe (ATL^8,9,15^).

The degree to which a face stimulus is individuated (i.e., seen as unique individual) versus categorized (i.e., seen as prototypical group member) depends on three factors: (i) the group or category a stimulus belongs to (e.g., human vs. robot; male vs. female), (ii) the perceiver’s perceptual experience with a stimulus group, and (iii) the perceiver’s motivation to see a stimulus as unique individual or prototypical group member (see^2^, for a review). This means that individuation requires the identification of a category, as well as sufficient motivation and experience to see a stimulus as an individual^2,18–20^. In contrast to individuation, categorization occurs spontaneously with little cognitive effort and does not require perceptual experience or motivation to discriminate a stimulus. Categorization focuses processing capacities on features that are category-diagnostic, which causes differences between categories (e.g., race, sex, age) appear more salient and differences within a category less salient^18,21,22^. Categorization consumes so little resources that category-relevant information can be extracted even under high task-load or despite being irrelevant to the task^23–28^. For stimuli to be individuated, perceivers first need to gain sufficient perceptual experience with the respective category through perceptual learning. Once perceptual experience has been acquired via prolonged exposure and in-depth processing, stimuli like cars, houses or dogs that usually do not cause face-typical processing, can show face-typical processing^8,13,14^. Dog experts, for instance, show an inversion effect when viewing dog pictures, whereas non-experts do not show such an effect^13^. Similar to perceptual experience, one’s motivation to individuate a stimulus is strongly influenced by its category status (here: whether a perceiver considers the stimulus as part of a relevant social in- or outgroup). For ingroup members, the motivation to see them as “individual” is high, attention is shifted to identity-diagnostic features (i.e., perceptual information that helps distinguish among group members), and individuation is facilitated. In contrast, engaging with members of a social outgroup lowers the motivation to see them as individuals, focuses attention on category-diagnostic features (i.e., perceptual information that most group members share), and is associated with attenuated individuation and increased stereotyping (e.g.^2,21^,). The motivation to individuate can be manipulated by varying the similarity between a perceiver and perceived stimuli, for instance, by changing physical features related to their appearance or by creating a certain belief about their social group affiliation^29^. Similar others (e.g., same race as the perceiver) are associated with increased individuation performance compared to dissimilar others (e.g., different race than the perceiver) due to better encoding of facial information (the “other race effect”; see^2,17^). The phenomenon is not specific to race but generalizes to all sorts of group affiliations, including gender, age and nationality; group affiliation seems to be such an omnipotent factor for individuation that even temporary (e.g., same university) or purely assigned (i.e., minimal group manipulations) group memberships induce measurable effects (see^2^; for a review).

Robots also have the capability to be categorically discriminated due to their category-diagnostic feature differences with humans, but challenge both the perceptual and motivational components of face perception. In terms of the perceptual component, robots are not part of our everyday lives yet, thus most humans have limited perceptual experience with this class of stimuli. Furthermore, robot faces might not be similar enough to human faces to fully engage face-typical processing because they often do not contain the same features as human faces (e.g., lack of eyebrows or disproportionally big eyes) and often do not display human-typical spatial relationships between facial features^30^. In terms of the motivational component, a robot’s lack of humanness might undermine one’s motivation to individuate them, as it might not be sufficiently similar to the perceiver. This would be in line with the observation that reducing someone’s perceived humanness through the act of dehumanization (e.g., in the case of racism) makes them seen as interchangeable and negatively impacts individuation^31,32^. Consequently, one way of increasing a perceiver’s motivation to individuate non-human stimuli is making them seem similar to the perceiver, thereby enhancing their chances of being perceived as an ingroup member^33^. In line with this statement, empirical studies have shown that leading perceivers to believe that nonhuman agents have humanlike capacities, made it easier for perceivers to individuate them (due to increased motivation, Almaraz et al., 2018). Research has also shown that although robots are generally seen as “outgroup”^34^, the likelihood that they are being perceived as “ingroup” increase when they display physical (e.g., gender^32,35,36^); or behavioral (i.e., mannerisms^31,33,37^;) signs of human-likeness. This suggests that the degree of a robot’s physical human-likeness has the potential to modulate the degree to which it is individuated. The degree to which a robot is individuated, in turn, should influence whether trust in human–robot teams is system-wide (in case individuation fails or is weak) or component-specific (in case individuation is in place). The current paper examines the first aspect of this argument, namely (i) whether robot faces can be individuated, (ii) how individuation of robot faces compares to human faces, and (iii) how individuation is impacted by the robot face’s physical human-likeness.

### Aim of study

Trust calibration is an important issue that needs to be addressed in human–machine teaming where non-human agents are often incorrectly perceived as a homogenous group with similar capabilities (i.e., SWT), rather than a group of individuals with different strengths and weaknesses (i.e., CST^1^;). To see robots as individuals, they need to be individuated (i.e., specific appearance has to be associated with a unique set of features^2^;), with other work showing different methods of individuation^38–40^. Here, we present two experiments that examine (i) the individuation of human versus robot faces (Experiment 1), as well as the effect of a robot’s physical human-likeness (high vs. low) on individuation performance (Experiment 2). In Experiment 1, we created families of robot stimuli that – similar to the Greebles—had one feature in common (e.g., long ears) but varied regarding other features (see Stimuli section for more information). We used this method of creating the stimuli, as opposed to using real-robot faces, because prior work has shown that nonhuman faces are perceived as more dissimilar than human faces, and are thus easier to discriminate^41,42^. Thus, in an attempt to create robot stimuli that are (i) equally similar to each other as human faces are, and (ii) contain a similar number and type of identity-diagnostic features as human faces do (i.e., people use perceptual features such as nose, mouth, and eye shape to discriminate human faces^2,11,43^), we are able to examine whether individuation differs between human and robot stimuli while controlling—to the best extent possible – for physical dissimilarities within stimuli groups. We expected to (i) replicate the “other race effect” for human stimuli (i.e., better individuation performance for white versus black face stimuli for white participants), and (ii) observe better individuation performance for human versus robot faces. This is based on the assumption that participants have (i) less perceptual experience with robots due to a lower exposure to robot vs. human faces in everyday life, and (ii) less motivation to individuate robots due to their potential outgroup status.

In Experiment 2, existing robot faces obtained from the ABOT database^44^, as opposed to digitally modified robot faces to examine whether differences in physical human-likeness would lead to differential individuation performance within the robot category. We expected robot stimuli with high levels of facial human-likeness to be better individuated than stimuli with low levels of facial human-likeness (i.e., robotic faces with many versus few human facial features; see methods of Experiment 2 for details).

## Experiment 1

The goal of Experiment 1 is to investigate whether humans can individuate robots based on their facial features and, if so, how their individuation performance compares to human “ingroup” and “outgroup” stimuli. To examine this question, participants completed an individuation task with one of three groups of faces: white human faces (i.e., racial “ingroup” for white participants), black human faces (i.e., racial “outgroup for white participants) or robot faces (i.e., robots); face type was manipulated between participants. The task consists of two phases: during the learning phase, participants were presented with five faces (white human, black human or robot faces) and had to learn to associate them with a certain identifier (letters A, B, C, D or E); in the recognition phase, participants were presented with the same faces again and had to pick the correct identifier via multiple choice. After their selection, participants received feedback, which helped them to improve individuation performance over time.

Given that “ingroup” status positively affects individuation, we expect individuation performance to be better for (i) white versus black human faces (the “other race effect”; see^2^), and (ii) human versus robot faces (as robots have “outgroup” status^34^).

### Methods and materials

#### Participants

253 white participants completed the experiment on Amazon Mechanical Turk via the turkprime platform (www.cloudresearch.com), with a median completion time of 14 min. Of the entire sample, 83 participants were assigned to the “black-human face” condition (BHF), 82 to the “white-human face” condition (WHF), and 88 to the robot face condition (RF). We excluded 12 participants (5 BHF, 4 WHF, 3 RF) due to extremely short or long completion time. Specifically, we excluded participants above or below five median absolute deviations from the median^45^, due to the heavily skewed distribution of completion time data, we decided against a mean- and SD-based approach. Additionally, we excluded sixteen participants (4 BHF, 12 RF) because of poor performance of below 30% accuracy (chance level: 20%), resulting in a final sample size of 225 participants (BHF: n = 74, 55 females, M age = 40.8; WHF: n = 78, 54 females, M age = 37.9; RF: n = 73, 53 females, M age = 40.1). Participants received $0.50 for their participation. Written informed consent was obtained from all participants prior to participation. This research and data handling complied with the Declaration of Helsinki and was approved by the Institutional Review Board at George Mason University.

#### Apparatus

Participants took the survey online on their own devices. The experiment was presented using Qualtrics (www.qualtrics.com). Stimulus presentation scaled with the size of the participant’s screen.

#### Stimuli

**Figure 1:**
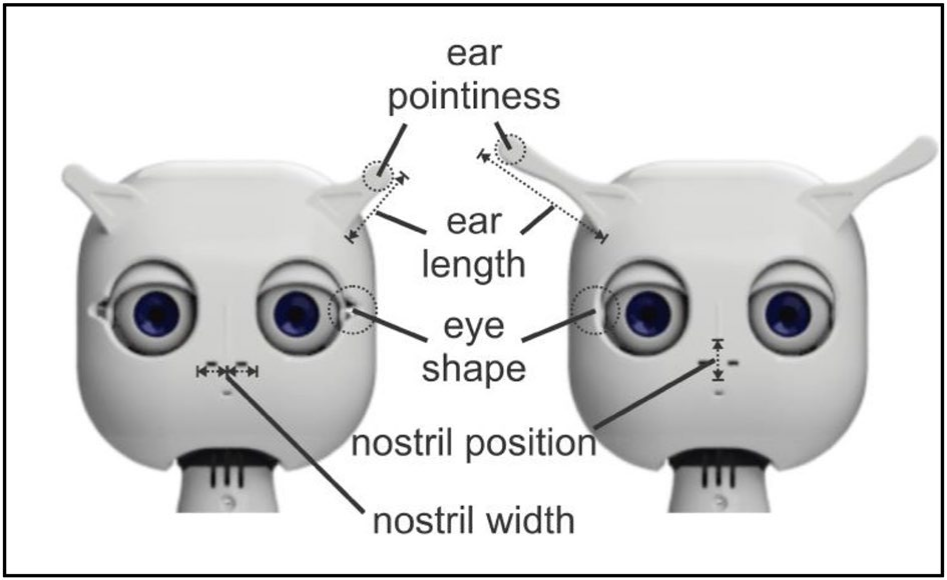
Robot face stimuli. Five robot faces differed in five parameters: ear pointiness, ear length, eye shape, nostril position, and nostril width. Each one of the five robots had one physical feature that stood out compared to the rest, which would allow participants to discriminate the robots.

Ten forward-facing human faces (5 black, 5 white) were randomly selected from the Chicago face database^46^. They were cropped using an oval shape in a way that background and hair were removed from the pictures. Five forward-facing robot faces were created and rendered using a standard 3D object manipulation software. The five faces differed in ear length (short to long), ear pointiness (pointy to round), nostril width (narrow to wide), and eye shape (round to oval; see Fig. 1). Similar to Gauthier and colleagues who used unique physical morphology features to identify Greebles of different families (e.g., Family X has long dunths), we aimed to have a unique facial feature that stood out for each robot (e.g., very pointy ears vs. ears that were more dull) that would allow participants to discriminate the robots based on their facial features. In our case, robot A had the most average features of all, robot B had the sharpest ears, robot C had the widest nose slits, robot D had the longest ears, and robot E had eye slits.

#### Task

**Figure 2:**
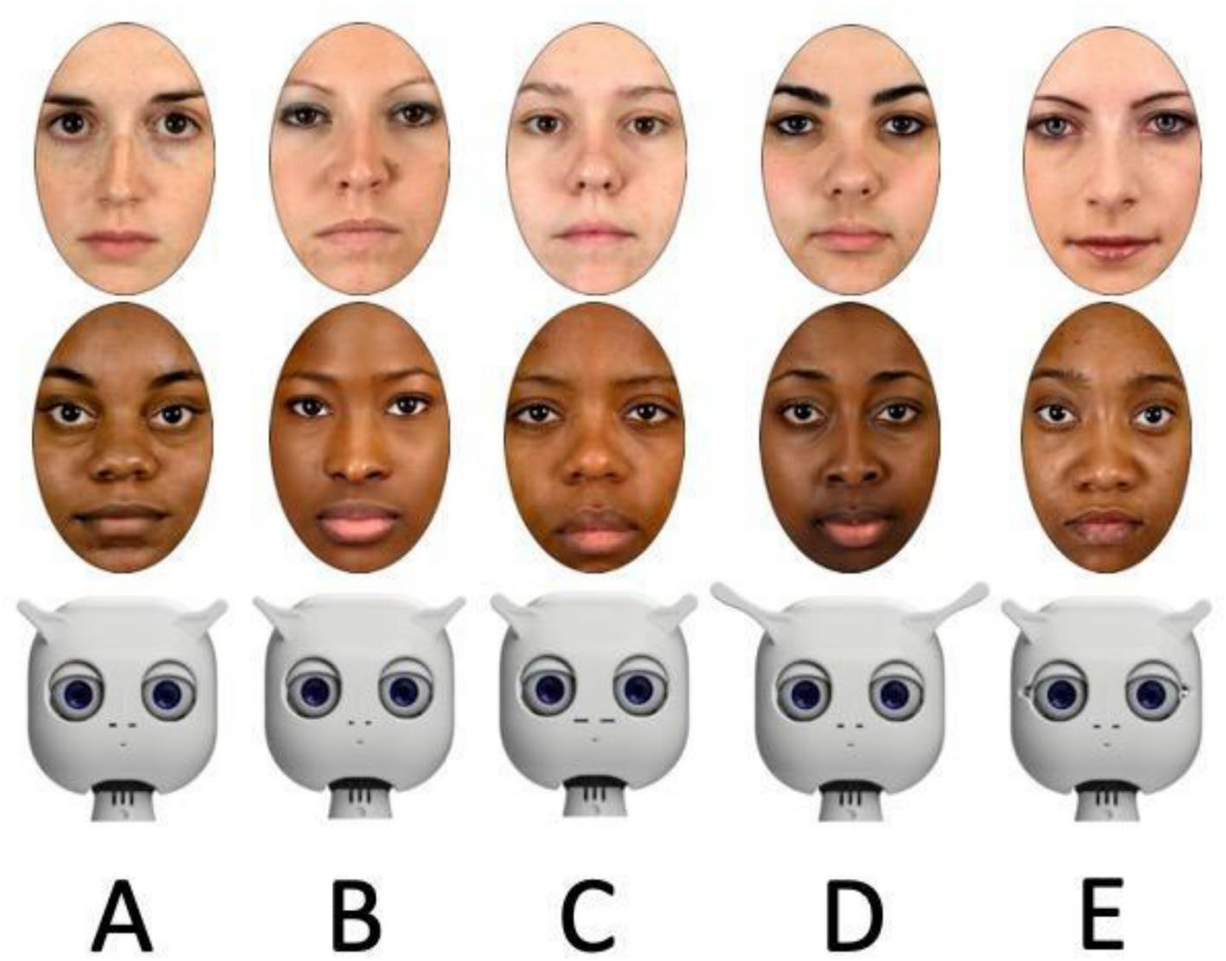
Stimuli used in Experiment 1. Participants saw five face stimuli based on the condition that they were assigned to (WFH: top, BFH: middle, or RF: bottom). Each face was associated with a unique letter, which participants needed to memorize (i.e., the memory task), and then they needed to recall which face was associated with which letter (i.e., perceptual task). The face stimuli were obtained from the freely available Chicago face database^46^.

The individuation task consisted of two sections: a learning phase (called “memory task”) and a recognition phase (called “perceptual task”). During the memory task, participants were provided with a cover story for all conditions (WHF, BHF, or RF): they were told that they would see an alien character together with a letter and that they needed to memorize which character was associated with which letter (A, B, C, or D). The cover story was provided for all the conditions to ensure that assigning a categorical label to the stimuli (i.e., human or robot) did not influence individuation performance. In the task, five faces (WHF, BHF, or RF) were presented together with a unique letter identifier (A, B, C, D, E); see Fig. 2 for the face-letter combinations for all three face types. Each face-identifier combination was presented only once, and the order of presentation was randomized across participants.

**Figure 3:**
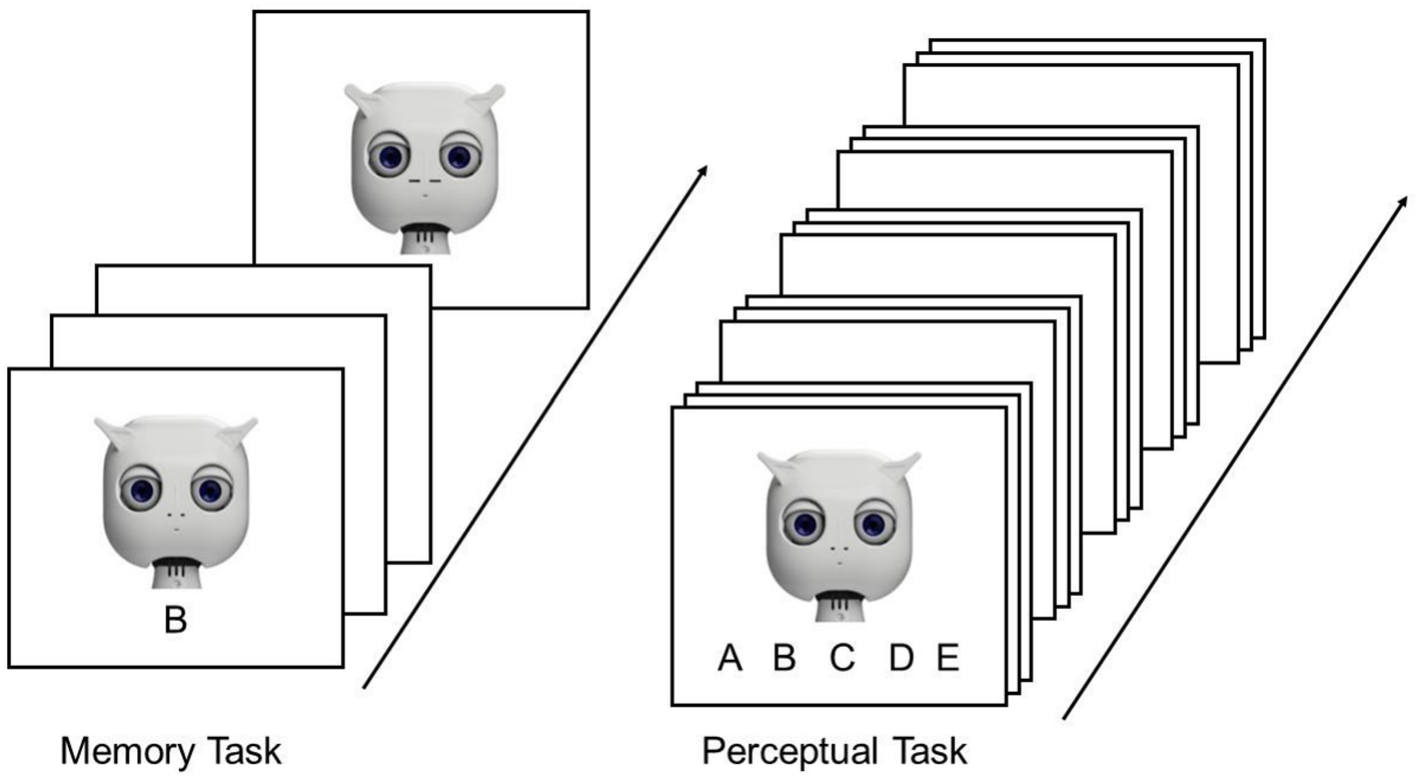
Trial sequence of the individuation task. The individuation task consisted of two parts: a *memory task* where participants saw and memorized five face-letter combinations (depicted here is the RF condition). After seeing all five face-letter combinations (letters A, B, C, D, E) were presented, the memory task started. During the *perceptual task*, participants completed five blocks of 20 trials each. During each trial, one of the faces from the perceptual task was shown again together with the letters A, B, C, D, and E presented underneath it. Participants were asked to pick the correct identifier and received feedback after they had made their selection.

During the perceptual task, participants were told that they were to see the same alien characters again and they needed to decide which alien character was shown (A, B, C, or D). The perceptual task consisted of five blocks of 20 trials each, resulting in 100 trials in total. During each block, each face was presented four times, and participants were instructed to choose the respective identifier (A, B, C, D, E) that has been paired with this phase during the perceptual task. The selection had to be made from a drop-down menu. The trial sequences of the perceptual task and the memory task are shown in Fig. 3.

#### Procedure

After clicking the link provided on MTurk, participants read a consent form. Once consent was provided, participants completed a demographics questionnaire. Next, they were instructed that the study involved a memory task and a perception task. In the memory task, they were provided the following cover story “Imagine that you are an anthropologist on an alien planet. After some field work and collecting pictures of the native species that inhabit the planet, you are tasked to study the aliens”. Next, subjects were told that they will view a series of 5 alien characters together with a letter (A, B, C, D, or E) and will be tasked to memorize which letter represents which alien. Participants were also instructed to take as much time as needed to memorize the alien-letter combination. After viewing all the alien-letter combinations in a randomized fashion, participants then started the “Perception Task”. In the perception task, participants were instructed that they were going to see the five aliens from the memory task again in a random order and their task was to decide which alien corresponded to which letter. After each trial, participants were provided feedback that showed their choice, whether it was correct or incorrect, and which was the correct answer if they answered incorrectly. After completing the “Perception Task”, participants were asked if they ran into any problems, were thanked for participating in the study, and were provided with a unique code that they entered on MTurk to receive payment.

#### Analysis

The analysis uses individuation performance as dependent variable. Individuation performance is defined as the percentage in which the correct letter was selected when presented with a face. As an omnibus test, we employed a repeated-measures mixed 3 × 5 ANOVA with *Target Face* as a between-participant factor (levels: WHF, BHF, and RF) and *Block Number* as a within-participants factor (levels: 1, 2, 3, 4, and 5). The ANOVA results were Greenhouse–Geisser-corrected where appropriate. For post-hoc testing, *t*-tests were employed. Analyses were conducted in R version 4.2.2^47^ and the afex package (version 1.2.1). All reported *p*-values were corrected for multiple comparisons using the Hochberg procedure^48^.

### Results

**Figure 4:**
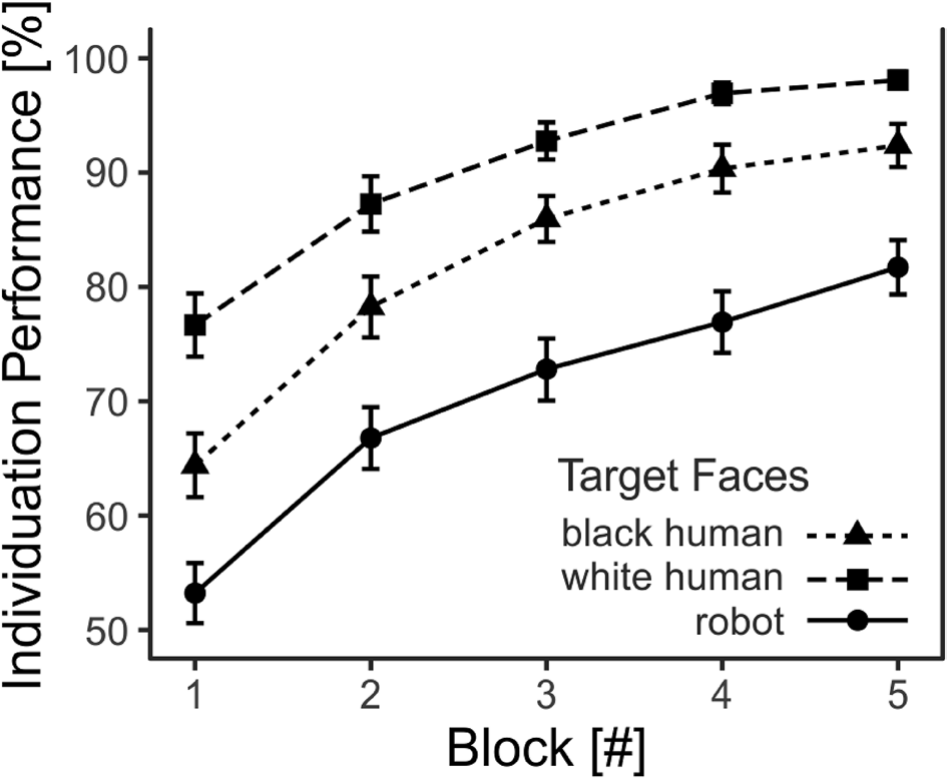
Individuation as a function of block number and type of face stimuli. Participants were able to individuate all the faces overall. The data also shows that white faces were individuated better than black faces. White faces and black faces, on the other hand, were individuated better than robot faces. Error bars represent SEM.

Individuation performance depended significantly on *Block Number* (*F*(2.3, 507.8) = 187.0, *p* < 0.0001, η_G_^2^ = 0.15) and *Target Face* (*F*(2, 222) = 26.8, *p* < 0.0001, η_G_^2^ = 0.18). *Block Number* and *Target Face* did not interact in their influence on individuation performance (*F*(4.6, 507.8) = 1.5, *p* = 0.21, η_G_^2^ < 0.01). Results were Greenhouse–Geisser corrected where appropriate. Post-hoc independent *t*-tests showed that participants performed best when individuating white faces, both in comparison to black *(t*(150) = 3.3, *p* = 0.001; *M*_*WHF*_ = 90.3%, *M*_*BHF*_ = 82.3%) and robot *(t*(149) = 7.3, *p* < 0.0001; *M*_*RF*_ = 70.3%) faces. Participants were also better in individuating black faces in comparison to robot faces (t(145) = 3.9, *p* = 0.0003). Another post-hoc dependent *t*-test validated that all participants learned how to individuate the faces throughout the experiment, as individuation performance for Block 5 was significantly higher than Block 1 *(t*(224) = 18.1, *p* < 0.0001; *M*_*Block 1*_ = 65.0%, *M*_*Block 5*_ = 90.9%) (Note that participants of all target face conditions were entered this analysis)*.* Results can be shown in Fig. 4.

### Discussion

Experiment 1 examined how the individuation of robot faces compared to the individuation of human faces (with “ingroup” or “outgroup” affiliation). Participants completed an individuation task with one of three target face groups: “ingroup” human faces (here: white), “outgroup” human faces (here: black), and rendered robot faces. Based on the assumption that (i) perceptual experience with is lower for robots compared to humans due to the lack of exposure to robots in everyday life, and (ii) “ingroup” status positively affects individuation performance, we hypothesized that participants would individuate “ingroup” human faces the best, followed by “outgroup” human faces and robot faces.

The results show that participants were able to individuate all three types of face stimuli as individuation performance significantly improved throughout the experiment for all stimulus groups. The results also showed the well-established “other race effect”, namely that white participants were better at individuating white versus black face stimuli (see^2^, for a review). In line with our hypothesis, individuation of the robot stimuli was also significantly worse than individuation of both the white and black human faces, potentially due to a combination of a lower perceptual experience with robot than human faces and a lower motivation to individuate robots compared humans as robots might be considered as an “outgroup”^34^. However, despite an effort to create robot face stimuli that are as perceptually similar (or dissimilar) as human faces, it cannot be ruled out that the reduced individuation performance for the robot faces compared to the two groups of human faces is at least partially due to a higher level of perceptual similarity among the rendered robot stimuli compared to the human stimuli.

To understand the impact of perceptual similarity to human faces on the individuation of robotic stimuli and in order to account for a higher level of perceptual variability in naturalistic robot faces (see^44^), Experiment 2 was conducted. Robot faces vary widely in terms of their physical human-likeness, such that some of them have only one human facial feature (usually eyes), whereas others have up to four human facial features (eyebrows, eyes, nose, mouth). Moreover, while human facial features are arranged in a standardized eyes-over-nose-over-mouth layout with little variation from human to human in terms of the spacing of these features, robot faces vary widely on this measure^49^. This indicates that the degree to which robot faces engage face-typical processes may depend on the degree to which they resemble human faces.

## Experiment 2

In Experiment 2, we investigated whether naturalistic robot stimuli’s perceptual similarity to human faces affects individuation performance. Specifically, we examined whether the number of a robot’s facial features—one of the main determinants of physical humanlikeness—modulates individuation of robots. To do so, and in contrast to Experiment 1, we pre-selected naturalistic robot faces based on the number of human facial features they displayed before participants performed the individuation task: robots with a low number of human facial features (only eyes = low face likeness; LFL) versus robots with a high number of human facial features (eyebrows, eyes, nose, and mouth = high face-likeness; HFL). Human face-likeness was varied within participants. We expected a robot’s human-likeness to modulate individuation, such that robots with HFL would be individuated better than robots with LFL.

### Methods and materials

#### Participants

In total, 192 participants of varying racial backgrounds completed the experiment on Amazon Mechanical Turk (www.mturk.com) via the turkprime platform (www.cloudresearch.com), with a median completion time of 11 min. Of the entire sample, 97 participants completed the experiment in the LFL), and 95 in HFL. We excluded 14 participants (7 in LFL, 7 in HFL) due to extremely short or long completion times with the same median-based approach reported for Experiment 1. Additionally, we excluded one participant (HFL) because of poor performance of below 30% accuracy (chance level: 20%), resulting in a final sample size of 177 participants (LFL n = 90, 55 females, M age 31.0; HFL n = 87, 60 females, M age 31.9). Participants received $0.50 for their participation. Written informed consent was obtained from all participants prior to participation. This research and data handling complied with the Declaration of Helsinki and was approved by the Institutional Review Board at George Mason University.

#### Apparatus

As with Experiment 1, participants partook in this experiment on their own devices. The experiment was presented using Qualtrics.

#### Stimuli

**Figure 5:**
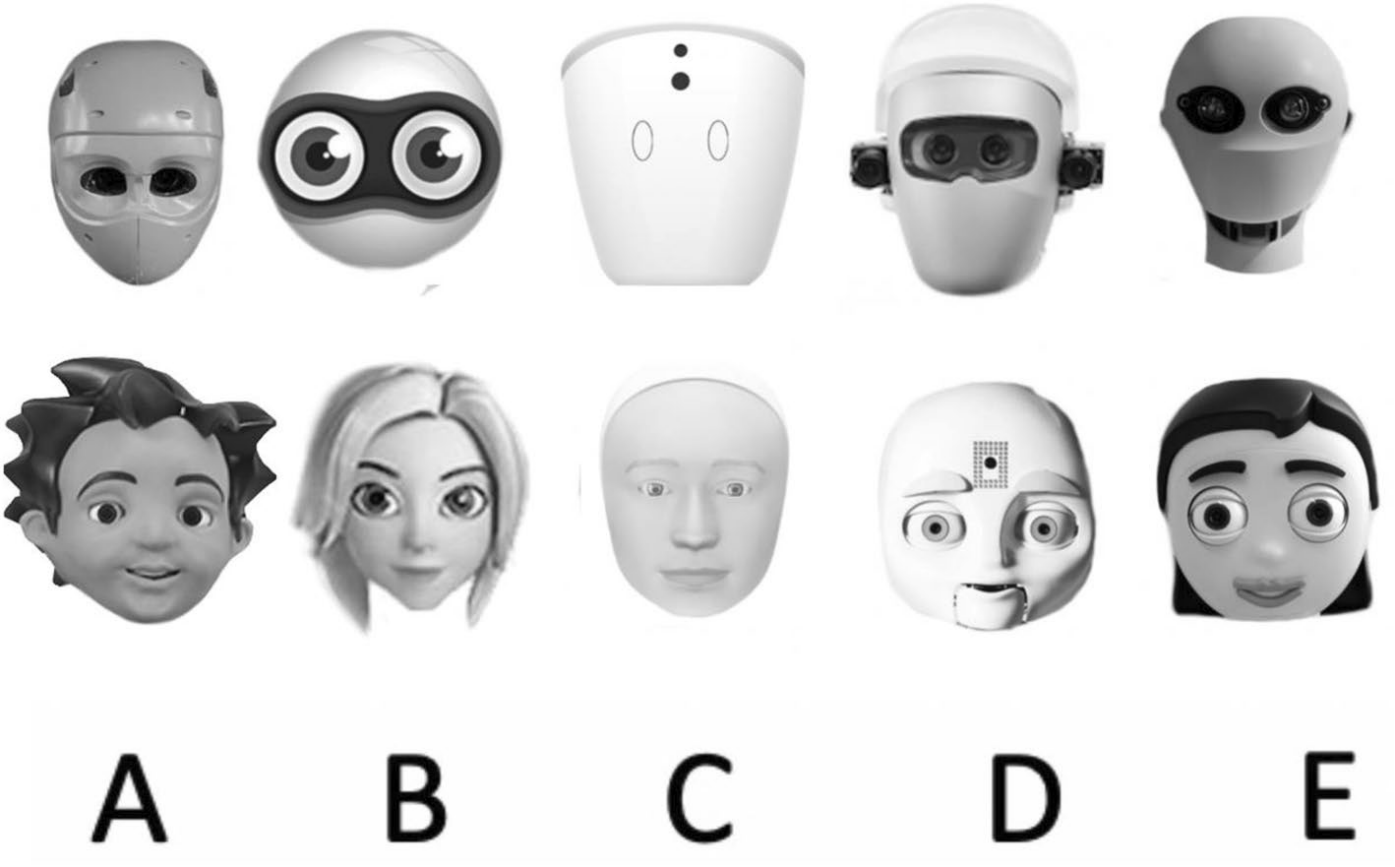
Stimuli used in Experiment 2*.* Participants were assigned to one of two conditions: Robots with low face likeness (here only eyes as human facial feature; see *top row*) and robots with high face likeness (here all human facial features: eyebrows, eyes, nose, mouth; see *bottom row*). The respective letter identifier is shown underneath the images.

A sample of ten robot faces was used in Experiment 2. They were a subset of 80 robot faces from the ABOT (Anthropomorphic robot database; Phillips et al., 2018) database and internet searches with the inclusion criteria being that faces (i) were white, (ii) contained eyes, (iii) were presented in frontal view and (iv) were fully visible (from the top of the head to the bottom of the chin). Of the resulting images, we quasi-randomly chose five that only contained eyes (LFL) and five that contained eyebrows, eyes, nose, and a mouth (HFL); see Fig. 5. If necessary, bodies and peripheral background areas were cropped so that only the robot’s face remained. All images were converted to gray scale and presented on a white background.

#### Task

The task in Experiment 2 was identical to Experiment 1. The only difference was that instead of human and robot faces, we used robot faces that had a high number of human facial features (i.e., high face-likeness; HFL) or a low number of human facial features (i.e., low face-likeness, LFL).

#### Procedure

The procedure in Experiment 2 was identical to Experiment 1.

#### Analysis

Analyses were analogous to Experiment 1. Specifically, we employed a repeated-measures mixed 2 × 5 ANOVA with *Target Face* as a between-participants factor (HFL vs. LFL) and *Block Number* as a within-participants factor (1, 2, 3, 4, and 5). Similar to Experiment 1, Individuation performance was used as dependent variable and was defined as the percentage in which the correct letter was selected when presented with a face. All reported *p*-values were corrected for multiple comparisons using the Hochberg procedure^48^.

### Results

Individuation performance significantly depended on *Block Number* (*F*(1.6, 286.6) = 76.0, *p* < 0.0001, η_G_^2^ = 0.12). *Target Face* (*F*(1, 175) = 5.7, *p* = 0.0184, η_G_^2^ = 0.02) was also significant, with participants performing significantly better when individuating HFL faces in comparison to LFL faces *(t*(175) = 2.4, *p* = 0.0184; *M*_*LFL*_ = 91.5%, *M*_*HFL*_ = 95.3%). *Block Number* and *Target Face* also interacted significantly in their influence on individuation performance (*F*(1.6, 286.6) = 3.6, *p* = 0.0369, η_G_^2^ = 0.01).

**Figure 6:**
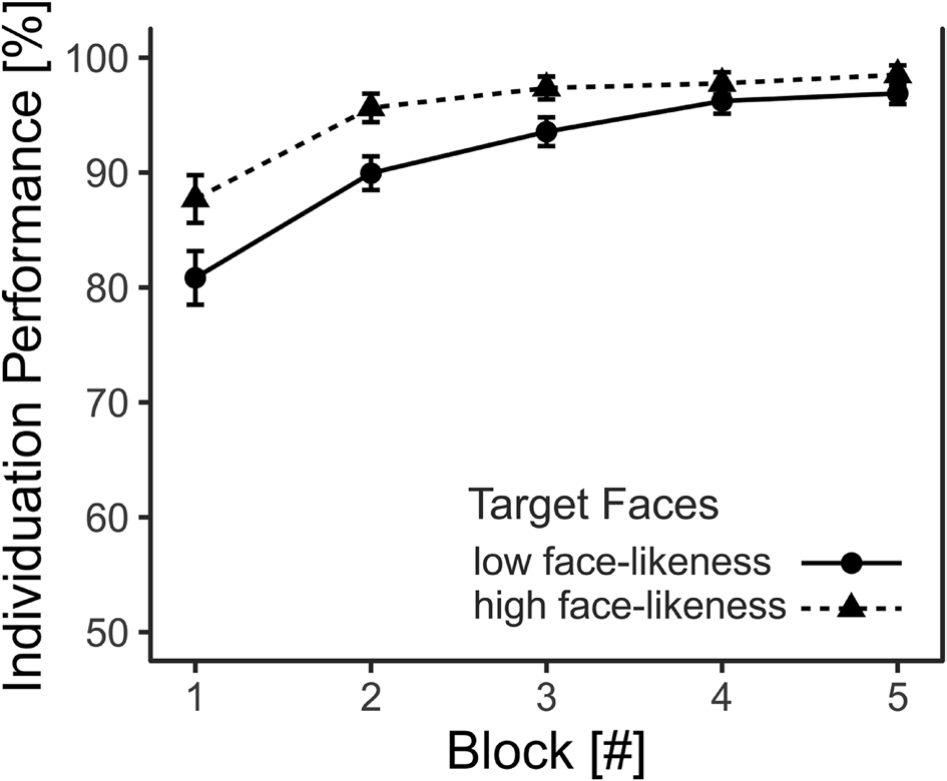
Individuation as a function of block number and the number of robot facial features. Results of Experiment 2 showed that participants were able to individuate all faces. No differences in individuation performance were detected between robot faces with one feature vs. robot faces with two features. Participants initially performed worse when individuating faces with one in comparison to four features; this difference diminished as the experiment progressed. Error bars represent the SEM.

Follow-up on the interaction showed that participants were worse at individuating LFL faces in comparison to HFL faces at the beginning of the experiment (Block 1: *t*(175) = 2.2, *p* = *0.0444; M*_*LFL*_ = 80.8%,* M*_*HFL*_ = 87.7%). However, this difference was not apparent at the end of the experiment (Block 5: *t*(175) = 1.3*, p* = *0.1*936*; M*_*LFL*_ = 96.9%, *M*_*HFL*_ = *98.5%*). Another post-hoc dependent *t*-test validated that all participants learned how to individuate the faces throughout the experiment as they individuated the faces in Block 5 better than Block 1 *(t*(224) = 10.1, *p* < *0.0001; M*_*Block 1*_ = 84.2%,* M*_*Block 5*_ = 97.7%) (Note that participants of all target face conditions entered this analysis). Results are depicted in Fig. 6.

### Discussion

Experiment 2 examined whether human face-likeness—as defined by the number of human facial features a robot stimulus contained—is a modulator of individuation of naturalistic robot faces. For that purpose, participants completed an individuation task with a group of robots that had either high face-likeness, as determined by a high number of facial features (eyebrows, eyes, nose and mouth) or low face-likeness, as determined by a low number of facial features (eyes only). We hypothesized that participants’ ability to individuate robot stimuli depended on the number of human facial features they contained, as resemblance to human faces is a predictor of face-typical processing^11,14,49,50^, and has the potential to increase one’s motivation to individuate a stimulus^2^.

Comparable to Experiment 1, Experiment 2 showed a significant improvement of individuation performance over the course of the five experimental blocks for all participants, indicating that both robot types (i.e., low and high human face-likeness) were individuated. Moreover, and in line with our hypothesis, participants showed better individuation performance for robot stimuli that displayed high human face-likeness than robots with low human face-likeness. Since it is reasonable to assume that both groups of robot stimuli were equally unfamiliar to participants, it is not likely that differences in perceptual experience accounted for the observed differences in participants’ individuation performance. It is possible, however, that robots with high face-likeness were perceived as more humanlike and as such were treated more like “ingroup” stimuli, which could in turn have led to a higher motivation to individuate them. This would be in line with the categorization-individuation model (CIM), which posits that similarity to one’s ingroup increases the motivation to individuate^2^. Interestingly, we also found an interaction between Block Number and Face Type, such that LFL robots were individuated to a lower extent than HFL robots at the beginning of the experiment, but this difference in individuation performance diminished at the end of the experiment. A similar interaction was not seen in Experiment 1, where we observed that learning occurred for all stimulus groups (i.e., perceptual discrimination improved for all stimuli), and the relative difference in individuation performance between stimulus groups remained constant throughout the experiment. Comparable to Experiment 1, it is possible that differences in perceptual similarity within the respective stimulus groups might account for the initial differences in individuation performance between HFL and LFL robots, such that the LFL stimuli might be inherently more similar to each other compared to HFL stimuli. Consequently, participants would have had to invest extra effort in order to be able to perceptually discriminate the LFL stimuli, which might have ultimately contributed to a better progression of individuation performance over time.

When descriptively comparing the results of Experiments 1 and 2, we noticed that overall individuation performance between the experiments differed, with better performance in Experiment 2 (two naturalistic robot stimulus groups) than in Experiment 1 (two human and one rendered robot stimulus group). This difference could indicate differential levels of perceptual similarity within different stimulus groups (i.e., in some groups, stimuli might be more similar to each other than in other groups). In order to explore this possibility, we ran a post-hoc control experiment to investigate how participants would subjectively judge the perceived similarity of the stimuli within the five different stimulus groups used in this study (three in Experiment 1 and two in Experiment 2). A detailed description of the methods, procedure and results of this control experiment can be found in the supplementary materials. The control experiment showed that participants subjectively perceived the stimuli used in Experiment 1 as more similar than those used in Experiment 2 (t(43) = 19.6, p < 0.0001, d = 2.95, 95% CId = [2.32, 3.60]), which would explain the overall better individuation performance in Experiment 2. Furthermore, the rendered robot stimuli were perceived as descriptively more similar than both groups of human stimuli in Experiment 1 (M_RF Similarity_ = 3.54 vs. M_BHF Similarity_ = 2.05 vs. M_WHF Similarity_ = 1.82); no subjective differences in similarity were found for the two groups of robot stimuli in Experiment 2 (M_LFL Similarity =_ 0.87 vs. M_HFL Similarity =_ 0.8). In sum, this shows that the overall level of individuation performance – in particular with the robot faces—was to a large degree determined by the perceptual similarity of the chosen stimuli: although perceptual learning occurred for both the rendered and the naturalistic robot stimuli, overall individuation performance was much better for the naturalistic than the rendered robot faces.

## General discussion

With engineering advances, it is becoming more common for humans to work with teams of robots instead of working with them in one-on-one settings^1,3,51^. However, to work effectively with robot team members, we need to be able to discriminate their individual abilities instead of seeing them as a uniform group^1^. This is known as trust calibration, and it is a critical issue in human–robot teaming as it allows us to appropriately allocate tasks to robot team members based on their actual skill sets. To achieve this, we need to be able to move robots from stimuli that are categorized (i.e., overgeneralization of individual capabilities), to stimuli that are individuated (i.e., discrimination of individual capabilities). The goal of the current study was to investigate how humans individuate robot faces in comparison to human faces that belong to either a social “ingroup” or “outgroup” (Experiment 1), and what impact a robot's physical human-likeness has on individuation performance (Experiment 2). In Experiment 1, we created rendered robot stimuli that shared a common feature but varied with regard to other characteristics (i.e., similar to the Greeble stimuli used by^16^). We expected better individuation of “ingroup” versus “outgroup” human faces, as well as human versus robot faces due to limited exposure to robots and their potential “outgroup” status^34^. In Experiment 2, we expected better individuation performance for robots with a high level of physical human-likeness (i.e., high number of human facial features) compared to those with a low level of physical human-likeness (i.e., low number of human facial features).

Results of Experiment 1 showed that participants were better at individuating the social ingroup compared to the social outgroup of human faces. This is consistent with prior work suggesting that humans usually have more perceptual experience with and are more motivated to individuate members of relevant social ingroups, which in turn positively affects individuation performance (e.g.^2,6^,). With respect to individuating robot stimuli, we demonstrated that humans are able to successfully individuate robots; however, this may occur to a lesser extent than both human ingroup and outgroup members—at least with rendered robot stimuli that are perceptually very similar. Differences in individuation performance between human and robot stimuli would also be in line with previous observations that robots are often perceived as outgroup members (e.g.^34^,), and could be driven by the fact that humans have lesser perceptual experience with robots compared to humans. This interpretation is in line with the CIM, which predicts that both a lack of perceptual experience and a reduced motivation to see “outgroup” members as individuals negatively impacts individuation performance^2,11,36,43^. Interestingly, the current results illustrate that humans with a social outgroup status were individuated better than robots, which – to the best of our knowledge—no work has reported before. While it is unclear to what extent perceptual vs. motivational factors account for the observed differences in individuation performance, it is conceivable that individuation is better for any human face compared to any robot face because participants may have more experience extracting configural information from human faces, rather than robot faces, which would allow them to shift attention to identity-level features and positively affect individuation (e.g.^2,11,43^,). It is also possible that robots are perceived as an even more distant social outgroup than humans belonging to a social outgroup leading to an even more reduced motivation to individuate robots than outgroup human stimuli^52^.

Results of Experiment 2 showed that robots that had high physical similarity to humans (i.e., high face-likeness; HFL) were individuated better than robots that had low physical similarity to humans (i.e., low face-likeness; LFL). Better overall individuation performance with HFL than LFL robot faces is possibly due to two main reasons. First, because HFL robot faces have a higher resemblance to human faces, it is possible that they were processed more configurally than the LFL robot faces, leading to better individuation performance. Second, it is possible that due to their higher human-likeness, HFL faces were associated with a higher level of social ingroup status than the LFL faces, which in turn could have increased participants’ motivation to individuate them^2^. The results of Experiment 2 also show that although LFL robot faces were individuated worse than HFL robots at the beginning of the experiment, no difference in individuation performance was detectable anymore at the end of the experiment, indicating that even robot faces with low levels of physical human-likeness can be individuated within a short learning period. This is consistent with previous work showing that people are able to individuate nonhuman agents^53^.

There are some open questions, that the presented experiments cannot answer. First, it is unclear to what extent differences in individuation performance were caused by perceptual versus motivational factors. Regarding perceptual factors, future studies will have to examine how differences in perceptual experience with humans versus robots might be offset by differences in perceptual (dis)similarities between human and robot faces (i.e., naturalistic robot faces seem to be more dissimilar to each other on average than human faces) when it comes to individuation performance. Thus, whereas higher perceptual experience with human faces compared to robot faces should benefit individuation of humans, it is conceivable that higher levels of perceptual dissimilarities among naturalistic robot faces should facilitate individuation of robot faces. Regarding motivational factors, it remains unclear whether robot stimuli are really seen as “outgroup” and as such are associated with lower motivation to individuate them. It is possible, for instance, that although naturalistic robot stimuli could easily be individuated based on their perceptual features but that a reduced motivation to see them as individuals still leads to suboptimal individuation performance. Second, although it is reasonable to assume that suboptimal individuation of robot agents would lead to system-wide trust and in turn suboptimal trust calibration, the presented data is not sufficient to draw conclusions related to trust in human–robot teams and should be examined in future studies.

With studies linking individuation to mind perception, our results also have important implications for the latter construct and potential effect on human–robot teaming. Mind perception is the tendency to ascribe emotions, beliefs, and intentions to non-human agents^54–56^. Notably, agents that are individuated well, are more likely seen as possessing “a mind”^53^. Since better individuation performance occurred with robots with more facial features in our experiment, this implies that robots with more facial features are more likely seen as having a human-like mind. Our results also imply that although trust calibration might initially be worse in human–robot than human–human teams potentially due to a reduced capability and / or motivation to individuate robotic agents, robot agents can be individuated and the degree to which this happens may depend on their physical human-likeness. This also suggests that robot team members’ human-likeness might particularly matter in short-term team interactions due to an initially reduced ability to distinguish between robots with different abilities. Given that individuation performance seems to improve rapidly with exposure, robots’ physical human-likeness might be less relevant for trust calibration in longer-term team interactions. However, in contexts where appropriate trust calibration is absolutely essential, for instance, because miscalibration would lead to physically, economically or otherwise negative consequences, robots with human-like facial features might still be the best choice as teammates. It is important to note that while the current work focuses on how individuation has the potential to calibrate trust in robot team members, alternate methods such as voice changes, speech patterns, and emotional expressions can help to calibrate trust appropriately in robot team members^38–40^.

## Conclusion

The purpose of the current study was to examine perceptual discrimination of social robots that differed in the number of facial features. In line with our hypotheses, we found that (i) humans are better individuated than robots and that (ii) robots with a high number of human facial features were more easily individuated than robots with a low number of human facial features. While this falls in line with recent HRI research examining the impact of facial features on face-typical processing, it is the first study to specifically examine the impact of facial features on individuation performance. The implication for HRI is that human–robot teams consisting of robots with more human-like facial features are likely to experience better team performance due to human operators being better at recognizing individual robots and their unique capabilities and accordingly assigning them to specific tasks. However, future work needs to investigate trust calibration in the context of individuation directly. The study also suggests that other known variables impacting face processing, such as a facial width-to-height ration (FWHR;^49,57^) or strengthened ingroup status (i.e., when a stimulus is seen as a closer group member^2,58,59^) might modulate individuation of robot agents and thus should be carefully considered when putting together human–robot teams. Lastly, our results also have important implications for mind perception—the tendency to ascribe human-like minds to non-human agents—since mind perception has been linked to individuation ability. Specifically, our results imply that robots with more humanlike features are more likely to be ascribed human-like internal states, such as emotions, beliefs, and intentions, all of which can improve the quality of a social interaction, increasing coordination between two partners and heightening prosocial tendencies during an interaction.

### Supplementary Information

Supplementary Information.

## Data Availability

Data is made available by the authors and can be found on the OSF page of the study: https://osf.io/48jnw/.
