# Supplementary material for: Perceptual discrimination in the face perception of robots is attenuated compared to humans
Source: Sci Rep. 2023 Oct 4;13:16708. doi: 10.1038/s41598-023-42510-6 (PMC10550918; doi:10.1038/s41598-023-42510-6)
Supplement: Supplementary file 1 — Supplementary Information. [file 41598_2023_42510_MOESM1_ESM.docx]

# SUPPLEMENTARY MATERIALS

As a post-hoc control experiment, we examined how people subjectively perceived the similarities between the stimuli of experiments 1 and 2.

## METHODS & MATERIALS

## Participants

A total of 44 participants (mean age 41.4 years; age range 18 – 81; 3 did not disclose age; 22 female, 22 male) were measured. Participants were recruited from countries with English as main language, including the United States, Canada, and the United Kingdom, through the plat-form Prolific (www.prolific.co). The sample size is based on a two-sided dependent t-test in G*Power (version 3.1.9.2, Faul et al., 2007); alpha = .05, 1 - beta = .9, and a medium effect size d = .5.

Apparatus

Participants completed the task on their personal computer running either Windows, Linux, or macOS. The task was programmed using PsychoPy (v2022.2.5; see Peirce et al., 2019) and presented online via the Pavlovia platform (www.pavlovia.org).

## Task and Procedure

The participants’ task was to rate the similarity of two stimuli at a time (‘How similar are the two agents presented above and below?’) on a visual analogue scale with five anchors (0 = 'very dissimilar', 1= 'dissimilar', 2 = 'somewhat similar', 3 = 'similar', 4 = 'very similar'). Each par-ticipant encountered all stimuli used in Experiments 1 and 2. Specifically, each participant rated the similarity of all possible dyads within each of the five stimulus groups (Experiment 1: black human faces, robot faces, white human faces; Experiment 2: low face likeness robot faces, high face likeness robot faces), resulting in ten trials for each group or 50 trials in total. The two faces that were presented each trial were shown above and below the visual analogue scale that ranged from left (0: ‘very dissimilar’) to right (4 = ‘very similar’) to avoid possible location-specific con-founds. Which face of a dyad was shown above or below was randomized.

## Analysis

To test whether stimuli in Experiment 1 were more similar than in Experiment 2, mean similarity ratings for Experiment 1 and 2 were averaged for each participant and compared with a dependent t-test. Effect size and associated interval were computed with the package bootES (version 1.2.1; the function bootES was used based on Cohen’s d, bias-corrected and accelerated confidence intervals, and R = 2000).

## RESULTS

Stimuli in Experiment 1 were substantially more similar than in Experiment 2; M Experiment 1 = .83 on a scale from 0 to 4, M Experiment 2 = 2.47 on a scale from 0 to 4, t(43) = 19.6, p < .0001, d = 2.95, 95% CId = [2.32, 3.60]. Descriptively, similarity was greatest between robot faces of Experiment 1 (3.54), followed by black and white human faces of Experiment 1 (2.05 and 1.82, respectively), and by low and high face like robot faces of Experiment 2 (.87 and .80, respectively).
